# Hospitalisations for chronic conditions among care experienced and general population children and young people: evidence from the Children’s Health in Care in Scotland (CHiCS) cohort study, 1990–2016

**DOI:** 10.1136/bmjpo-2024-002705

**Published:** 2024-10-02

**Authors:** Mirjam Allik, Edit Gedeon, Marion Henderson, Alastair Leyland

**Affiliations:** 1 University of Glasgow, Glasgow, UK; 2 University of Strathclyde, Glasgow, UK

**Keywords:** Diabetes, Adolescent Health, Child Health, Social work, Epidemiology

## Abstract

**Objective:**

There is limited evidence on how the physical health of children and young people (CYP) who are care experienced (eg, in foster or out-of-home care) compares to the general population. UK research suggests that the prevalence of some chronic conditions may be similar for these groups.

**Design:**

We undertook longitudinal population-wide data linkage of social care, prescription and hospitalisation records for care experienced and general population CYP born 1990–2004, followed from birth to August 2016. We compared prevalence estimates for asthma, diabetes (type 1) and epilepsy between the cohorts and used Poisson and survival models to estimate the association between social care and hospitalisations for these conditions.

**Results:**

Care experience was not associated with a higher prevalence of asthma and diabetes, but epilepsy was more prevalent. Care was associated with increased hospitalisation rates for all three conditions, particularly for males. HRs for hospitalisations were highest before and after care and lower while the child was in care, for diabetes these were, respectively 1.88 (95% CI 1.28 to 2.77), 2.40 (95% CI 1.55 to 3.71) and 1.31 (95% CI 0.91 to 1.88) for care experienced CYP compared with general population.

**Conclusions:**

Hospitalisations for chronic conditions are higher among care experienced CYP, particularly for males, and outside care episodes. Families with children with chronic conditions should be offered support to manage these conditions and help keep families together. Higher hospitalisations after care suggest that care leavers should be provided more support to help manage their health.

**WHAT IS ALREADY KNOWN ON THIS TOPIC**

- Evidence on the physical health of children and young people (CYP) who are care experienced (eg, foster or out-of-home care) is limited.
- UK research has found a higher prevalence of some physical health conditions (epilepsy) among care experienced CYP compared the general population but not others (asthma, diabetes).

**WHAT THIS STUDY ADDS**

- Prevalence of asthma and diabetes is similar among care experienced and general population CYP, while prevalence of epilepsy is higher among the care experienced group.
- Despite similar prevalence, care experienced CYP are more likely to be hospitalised for all three conditions.
- Hospitalisation rates are highest among males and outside care placements, such as before entering and after leaving care.

**HOW THIS STUDY MIGHT AFFECT RESEARCH, PRACTICE OR POLICY**

- Families with CYP living with chronic conditions should be offered more support to manage these conditions to help them stay together where it is safe to do so.
- Care leavers should be provided holistic (emotional, social, practical and financial) support beyond ages 16–18 to help young people start independent life and prevent hospitalisations for chronic illnesses.

## Introduction

 Much of the research on the health of children and young people (CYP) who are care experienced (also referred to as ‘looked-after’ children or children in foster or out-of-home care) has focused on mental health, neurodevelopmental conditions and emotional-behavioural well-being.^1^,^4^ Given the higher mortality in adulthood,^5^ relatively little is known about how the physical health of this group of CYP compares to the general population. Studies have reported worse dental health among those in care,^3 6^ but evidence is sparce with regard to other physical health conditions.^2 3 7^ To fill this gap, we focus on differences in hospitalisation rates between care experienced and general population CYP for asthma, diabetes (type 1) and epilepsy, the three most common chronic conditions leading to hospitalisation among CYP in Scotland and the rest of the UK.

The focus on hospitalisations for the three conditions is highly relevant for health and social care policy as unplanned in-patient admission rates are high among children with chronic conditions. In England, the three conditions account for around 94% of emergency admissions among children with long-term conditions and are used as one of the performance indicators for the National Health Service.^8^

UK studies among those aged 18 or younger have found no association between receiving childhood social care and asthma or diabetes but noted an increased prevalence of epilepsy.^2 7^ US studies have more frequently reported a higher prevalence of physical ill health among foster children, including respiratory and other chronic conditions .^9 10^

There is more evidence on the effects of adverse childhood experiences (ACEs) on physical health, consistently showing that these have a negative impact on the developing immune system and can lead to the development of chronic inflammatory conditions that may last for a lifetime.^11^ Most children who experience ACEs will not enter social care, but all care experienced children will have experienced some adversity in their childhood. Often they experience this at very high levels, including combinations of multiple adversities (such as domestic violence, parental substance misuse and mental ill health), leading to the negative impacts on health manifesting earlier in life and at higher intensity.^12^ Currently, the studies linking ACEs to adverse health mostly refer to health in adulthood and life course patterns and childhood health of those experiencing ACEs (or specifically childhood social care) remain almost undocumented.^13^ Health inequalities are likely to increase with age^14^ and might not be evident in childhood.

Our study is unique as it looks at whether inequalities in health, related to adversity, are already evident in childhood. We report prevalence estimates of asthma, diabetes (type 1) and epilepsy and provide the first longitudinal evidence in the UK on how hospitalisation rates for these conditions compare between care experienced and general population CYP. As the previous literature generally suggests that ACEs and childhood social care are associated with worse health, we hypothesise that compared with the general population, care experienced CYP have a higher prevalence of physical ill health and are more frequently hospitalised for the three chronic conditions studied here. In addition to the above, we also investigate whether hospitalisations among the care experienced cohort are more common before, during or after care. Here, we have little past evidence to guide our hypothesis and assume social care to be protective against adverse health events, such as hospitalisations. Therefore, we hypothesise that hospitalisation rates are higher before and after care relative to the general population, but we do not expect higher hospitalisation rates while children receive social care.

## Data and methods

We use the population-wide longitudinal Children’s Health in Care in Scotland (CHiCS) cohorts described previously for this analysis.^15 16^ CHiCS links administrative data on social care, births, hospitalisations and prescriptions to compare the health of care experienced children to children in the general population at the population level. The cohorts include 13 830 care-experienced and 649 771 general population CYP who were in school in Scotland in 2009 (born between 1990 and 2004). The models presented here included the subset of children with a chronic condition: 96 710 for asthma, 5620 for diabetes and 3286 for epilepsy. The hospitalisation and care records for the cohort members are followed from birth up to 31 July 2016 or death if before that date.

Our data include age (in months) for each hospitalisation and, for care experienced children, also the age (in months) at which their care episode started, changed care placement (such as between different types of care) or left care. This means that we can place each hospitalisation to a specific time point in a child’s life course and journey through care, that is, we know which hospitalisations occurred before, during or after leaving social care.

There are, however, some limitations to this. While most children who enter care only have one episode of care, that is, they enter care, are in care and then leave care, some experience multiple episodes. Our data does not capture episodes of care which ended before April 1st 2008, meaning that a small proportion of early episodes of care are excluded. See online supplemental file 2 for further details.

Also, if a child leaves care before the age of 16, it is possible for them to re-enter care. In our data, the majority of children who left social care were aged 16 or older at the end of our study, meaning that they will not re-enter care. Others may have re-entered care after the end of the follow-up period. This means that, in our data, the category of children who have left care is heterogeneous, though most will be young adults who have permanently left care.

We first use Poisson models to predict hospitalisation rates (planned and emergency) during the study period. In these models, care experience is measured with a binary variable indicating if the child has ever been in care. Person-years in the study are used as an offset to account for varying lengths of follow-up. These models do not use information about when in a child’s life course and journey through care the hospitalisations occur and only compare overall hospitalisation rates between the two cohorts.

We then use repeated events survival analysis to estimate the effects of the covariates on each individual hospitalisation, using attained age (in months) as the timescale. These models use information about when in a child’s life course and journey through care the hospitalisations occur. The child’s journey through social care is included as a time-varying covariate and we can separately estimate the effects of before, during and after the end of care placement, with the reference category being children who have never been in care.

Our definitions of asthma, diabetes and epilepsy are based on previous research,^17 18^ using the International Statistical Classification of Diseases and Related Health Problems 9th and 10th Revisions (ICD-9/10) and the British National Formulary (BNF). Definitions of prevalence used are as follows:

- Asthma—at least one hospitalisation for J45–J46 (493 for ICD-9) or two prescriptions for BNF sections 3.1, 3.2 or 3.3 within 12 consecutive months.
- Diabetes—at least one hospitalisation for E10–E14 (250 for ICD-9) or one prescription for BNF section 6.1. Both types are combined as we were not able to distinguish between type 1 and 2 diabetes in the earliest hospitalisations data (pre-1996), but data since 1996 suggest 90% of cases are type 1 diabetes in both cohorts.
- Epilepsy—at least one hospitalisation for G40–G41 (345 for ICD-9). Prescriptions for antiepileptic medications were excluded as these are increasingly used to treat conditions other than epilepsy.^19^ This definition excludes psychogenic non-epileptic seizures (PNES) which are of psychological causes, such as severe stress or trauma and coded in ICD-10 as dissociative disorders (F44).^20^ However, a misdiagnosis of epilepsy for patients with PNES is possible and, therefore, cation is needed when interpreting the results.

Deprivation is measured at the small-area level (datazones, population mean=815, SD=275) using population-weighted quintiles of the Scottish Index of Multiple Deprivation (SIMD). The population-weighted quintiles were calculated such that each quintile includes approximately 20% of the total Scottish population. We used home datazone at birth and the closest available 2004 SIMD when this was present (88% cases for both cohorts). This means that for the majority of our care experienced children we use the socioeconomic status of the birth parents and not that of the carers. For children born outside Scotland, we used the 2009 SIMD of the area of residence listed on the Pupil Census, which might indicate the area deprivation of the carer. A twofold urban–rural classification (at birth) at datazone level was used to identify area type (urban —settlements of 10 000 or more people; rural—all other areas).

A binary indicator for comorbidities was defined using hospitalisation records and included life-limiting and life-threatening conditions, as defined by past research,^21^ spina bifida, cleft lip and cleft palate, cerebral palsy and other paralytic syndromes, and other congenital malformations not included among life-limiting conditions. A binary indicator of whether the child was assessed disabled comes from the Pupil Census. The year of birth (in Poisson models) and a three-category birth cohort indicator (event history models) were also included. In event history models, birth cohort, comorbidities and disability were included as strata.

### Sensitivity analysis

As disability has the highest proportion of missing values (table 1), we tested models excluding disability to increase sample size. Additional models for children with birth records included mother’s age and parent’s employment status at birth (see online supplemental table S-1 for variable summaries and definitions). We estimated event history models where the time in care was split into four care placement types: (1) at home under a supervision order, (2) in kinship care, (3) in foster care and (4) in residential care. We explored if the effect of care type varied by sex (as assigned at birth) and if the effect of sex varied by age group.

**Table 1:** Prevalence estimates, hospitalisation numbers and distribution of variables by chronic conditions and cohort

	Asthma				Diabetes				Epilepsy			
	GPC		CEC		GPC		CEC		GPC		CEC	
	N	%	N	%	N	%	N	%	N	%	N	%
N Children/prevalence	94 700	14.6	2242	16.2	5501	0.8	142	1.0	3152	0.5	160	1.2
N Hospitalisations/mean	33 030	0.35	1055	0.47	11 167	2.0	628	4.5	9633	3.1	643	4.0
Cohort descriptives												
Female	43 376	45.8	1057	47.1	2961	53.8	89	62.7	1477	46.9	58	36.3
Male	51 324	54.2	1185	52.9	2540	46.2	53	37.3	1675	53.1	102	63.8
Deprivation												
1—Low	14 628	15.4	57	2.5	897	16.3	17	12.0*	484	15.4	12	7.5
2	16 742	17.7	146	6.5	1002	18.2			541	17.2	12	7.5
3	18 171	19.2	270	12.0	1049	19.1	22	15.5	592	18.8	18	11.3
4	19 882	21.0	491	21.9	1205	21.9	27	19.0	657	20.8	35	21.9
5—High	25 269	26.7	1254	55.9	1347	24.5	73	51.4	877	27.8	81	50.6
NA	8	0.0	24	1.1	1	0.0	3	2.11	1	0.03	2	1.25
Urban	64 257	67.9	1747	77.9	3647	66.3	101	71.1	2121	67.3	115	71.9
Rural	30 435	32.1	471	21.0	1853	33.7	38	26.8	1030	32.7	43	26.9
NA	8	0.0	24	1.1	1	0.0	3	2.11	1	0.03	2	1.3
Comorbid	5906	6.2	201	9.0	1380	25.1	45	31.7	1161	36.8	51	31.9
Not	88 794	93.8	2041	91.0	4121	74.9	97	68.3	1991	63.2	109	68.1
Disabled	2729	2.9	154	6.9	445	8.1	14	9.9	953	30.2	48	30
Not	91 971	97.1	1866	83.2	5056	91.9	106	74.6	2199	69.8	87	54.4
NA	0	0.0	222	9.9			22	15.5		0	25	15.6
Birth cohort												
(1990–1996)	25 420	26.8	798	35.6	2009	36.5	75	52.8	1082	34.3	69	43.1
(1996–2000)	32 256	34.1	757	33.8	1918	34.9	45	31.7	1087	34.5	44	27.5
(2000–2004)	37 024	39.1	687	30.6	1574	28.6	22	15.5	983	31.2	47	29.4
N complete observations	94 692	99.99	2018	90.0	5500	99.98	120	84.5	3151	99.97	135	84.4
(Used in models, excludes all missing values)												

* Due to statistical disclosure control, we had to combine the deprivation deciles 1 and 2 for care experienced children with diabetes. This has only been done in the table 1 and in the models we use the exact deprivation decile.

CEC, care experienced children; GPC, general population children; NA, not applicable.

### Patient and public involvement

We collaborated with the Centre for Excellence for Children’s Care and Protection when planning this research project and regularly consulted with the study advisory group (including representatives from children’s charities and public authorities responsible for the welfare of children and care experienced children) to help guide and contextualise the research.

## Results

The estimated prevalence of asthma and diabetes is similar in the two cohorts of care experienced and general population CYP, while the prevalence of epilepsy is twice as high among care experienced people (table 1) (see online supplemental table S-2 for a comparison to population statistics.) The mean number of hospitalisations per child with a condition is higher for care experienced children, particularly for diabetes and epilepsy.

There are more males among those with asthma and epilepsy and more females among those with diabetes (table 1). Care experienced CYP are more likely to be from deprived urban areas, and to experience other comorbidities and disabilities. Common disabilities among care experienced CYP are social, emotional and behavioural problems (39%, from those with a disability) and learning disabilities (20%).^16^

**Figure 1:**
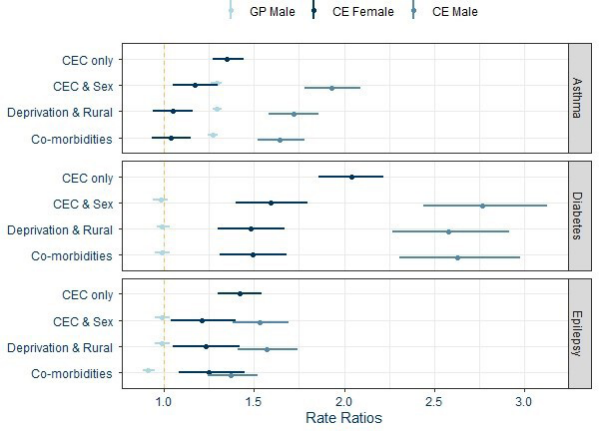
Rate ratios and 95% CI for general population males and care experienced females and males from Poisson models (Ref: general population females). Models: CEC only—care experience only included in the model; CEC and sex—sex added to the previous model; Deprivation and rural—added to the previous model; Comorbidities—added to the previous model. CEC, care experienced children; GP, general population.

Poisson models show that care experience increases the rates of hospitalisations for all three chronic conditions (figure 1). The association of care with the number of hospitalisations is most notable for diabetes (rate ratio, RR 2.04; 95% CI 1.86 to 2.22) and lowest for asthma (1.35; 95% CI 1.27 to 1.44). Including sex shows that while both care experienced males and females have higher hospitalisation rates compared with general population females, the RR is higher for care experienced males.

In the adjusted models, the RRs for care experienced females are attenuated for asthma (RR 1.04; 95% CI 0.93 to 1.15) and diabetes (1.49; 95% CI 1.31 to 1.68) hospitalisations (figure 1 and table 2). For epilepsy hospitalisations, the inclusion of comorbidities reduces the RRs for both general population and care experienced males.

**Table 2:** Poisson model rate ratios (RR) and 95% CI estimating the number of hospitalisations (person-years used as offset)

Variable	Asthma			Diabetes			Epilepsy		
	RR	95% CI		RR	95% CI		RR	95% CI	
		Low	High		Low	High		Low	High
Intercept	0.01	0.01	0.01	0.10	0.10	0.11	0.14	0.13	0.14
Ref: female GP									
Male GP	1.27	1.24	1.30	0.99	0.95	1.03	0.91	0.88	0.95
Care experienced female	1.04	0.93	1.15	1.49	1.31	1.68	1.25	1.08	1.45
Care experienced male	1.64	1.52	1.78	2.63	2.31	2.98	1.37	1.24	1.52
Deprivation (ref 1—Low):									
2	1.13	1.08	1.18	1.02	0.95	1.09	1.02	0.95	1.10
3	1.12	1.07	1.16	1.10	1.03	1.17	1.13	1.06	1.21
4	1.37	1.32	1.43	1.25	1.18	1.33	1.00	0.94	1.07
5—High	1.54	1.48	1.59	1.35	1.27	1.43	0.96	0.90	1.02
Rural (ref urban)	0.93	0.91	0.95	0.88	0.85	0.92	0.96	0.92	1.00
Comorbid	1.52	1.46	1.57	0.96	0.92	1.01	1.29	1.24	1.34
Disabled	1.12	1.06	1.18	0.90	0.83	0.96	2.21	2.12	2.31
Year of birth	1.00	1.00	1.01	1.02	1.01	1.02	1.07	1.06	1.07
N Children	96 710			5620			3286		

GP, general population.

In sensitivity analysis, we removed disability from the models but this had no substantial impact on the results (not shown). For children with birth records, we included mother’s age and parent’s employment status at birth in the models (online supplemental table S-3). This had a marginal impact on the RR for care experience and sex.

**Figure 2:**
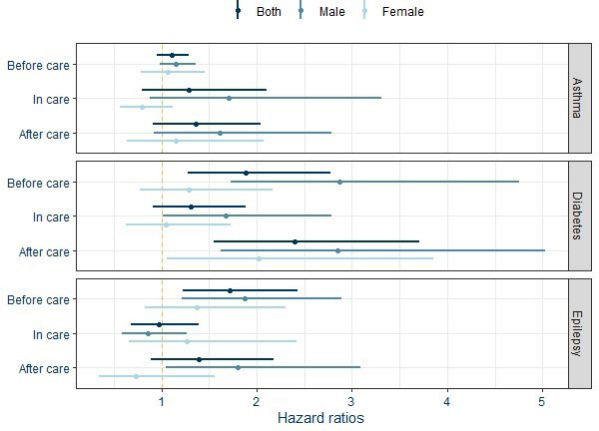
HRs and 95% CI for the effects of before, during and after care on hospitalisations for both sexes and by sex for the three conditions, fully adjusted models. Results for both sexes in table 3 and by sex in online supplemental tables S-5a, S-5b.

The repeated events survival models (table 3 and figure 2) show that the HRs of diabetes and epilepsy hospitalisations are respectively 1.88 (95% CI 1.28 to 2.77) and 1.72 (95% CI 1.22 to 2.43) for care experienced children before they enter care compared with those who never entered care. For diabetes, HR for hospitalisations after care is 2.4 (95% CI 1.55 to 3.71) compared with those who were never in care. For all conditions, the CIs for the HR for the period when the child was in care include one. The sensitivity analysis including mothers age and parent’s employment at birth had a marginal impact on the HR (online supplemental table S-4).

**Table 3:** HR and 95% CI for repeated events event history models for hospitalisations for asthma, diabetes and epilepsy

Variable	Asthma			Diabetes			Epilepsy		
	HR	95% CI		HR	95% CI		HR	95% CI	
		Low	High		Low	High		Low	High
Reference: never in care									
Before care	1.11	0.95	1.29	1.88	1.28	2.77	1.72	1.22	2.43
In care	1.29	0.79	2.10	1.31	0.91	1.88	0.97	0.68	1.39
After care	1.36	0.91	2.04	2.40	1.55	3.71	1.39	0.89	2.18
Male	1.28	1.20	1.36	1.02	0.91	1.14	0.93	0.82	1.04
Deprivation (ref 1—Low):									
2	1.13	1.03	1.24	1.02	0.88	1.18	1.03	0.85	1.25
3	1.12	1.02	1.23	1.09	0.93	1.29	1.14	0.93	1.41
4	1.37	1.25	1.50	1.25	1.07	1.47	1.01	0.84	1.21
5—High	1.53	1.40	1.68	1.35	1.14	1.61	0.98	0.81	1.17
Rural (ref urban)	0.93	0.87	0.99	0.88	0.79	0.98	0.95	0.83	1.07
N of hospitalisations		33 983			11 676			10 218	
N children		96 710			5620			3286	

Strata include co-morbidities, disabilities, and birth cohort.

The models in table 3 were also run separately for males and females (online supplemental tables S-5a, S-5b and figure 2). HRs were generally higher for males before, during and after care, with notable differences for diabetes and epilepsy hospitalisations. The HRs for diabetes hospitalisation are 2.87 (95% CI 1.73 to 4.76), 1.67 (95% CI 1.01 to 2.78) and 2.85 (95% CI 1.62 to 5.03), respectively, before, during and after care for males, but 1.29 (95% CI 0.77 to 2.17), 1.05 (95% CI 0.63 to 1.73) and 2.02 (95% CI 1.06 to 3.85) for females.

When the time spent in care was split into four care types (at home, in kinship, foster and residential care), the highest HRs were for placements at home or in residential care and the lowest for kinship and foster care (online supplemental table S-6). However, the CIs are wide and include one in all models.

We explored whether the effect of sex changes with age by testing interactions with age groups using a cut-off at 12 years (online supplemental table S-7). In the case of asthma, males had substantially higher hazards of hospitalisations before age 12 but much lower hazards after age 12. For diabetes, males had higher hazards before age 12 but there were no sex differences after age 12. Age did not interact with sex for epilepsy hospitalisations.

Finally, separate models for the care experienced cohort (results not shown) showed that the RRs of many covariates (deprivation, comorbidities) were low, and these factors may have a limited impact on hospitalisations among children who receive social care. However, the sample sizes of these models are small and the evidence is not robust.

## Discussion

Consistent with two other UK-based studies,^2 7^ we show that the prevalence of asthma and diabetes (type 1) is similar among care experienced CYP compared with the general population but care experienced CYP have a higher prevalence of epilepsy. The difference in epilepsy prevalence between the cohorts may be related to epilepsy being associated with neurodevelopmental conditions (eg, ADHD and autism)^22^ that are more prevalent among care experienced people.^2^

As the causes of type 1 diabetes, many cases of epilepsy and asthma are not fully understood and are likely independent from the causes of receiving childhood social care, it is not surprising that few differences in their prevalence have been found among care experienced and general population CYP. However, hospitalisation rates for these conditions (while dependent on the severity of the condition) are affected by the socioeconomic and family environments, access to primary care, and any support CYP and their families receive. For example, children from deprived backgrounds have poorer management of and more frequent hospitalisations for epilepsy, asthma and diabetes.^23^,^26^ We show that care experienced CYP have more hospitalisations for all three chronic conditions. This is similar to recent findings from Denmark showing that ACEs are related to higher hospitalisation rates across different diagnostic criteria from birth to early adulthood.^13^ More frequent hospitalisations for chronic conditions in childhood could lead to a more rapid progression of an illness and worse health outcomes as an adult, supporting previous findings that have associated ACEs with more chronic health issues in adults.^27^

HRs for hospitalisations among care experienced CYP for diabetes and epilepsy were highest outside care episodes, that is, before entering and after leaving care. Higher hospitalisation rates prior to entering care may indicate weaker service engagement at the general practice level. This weak engagement might itself stem from social disadvantage and difficulties some families face accessing timely healthcare (eg, inflexible working hours, poor access, parent’s ill health), but also from poor doctor–patient relationships.^28^ A lack of trust in the medical profession has been linked to ACEs^29^ and prevent some CYP and their families from seeking help.

Our results showing that hospitalisations tend to be lower while children are in care are encouraging, indicating that the support CYP receive while in care may help overcome some of these barriers and potentially improve health. Unfortunately, diabetes and epilepsy hospitalisations increase after care placements end. For CYP who are under the age of 16 and return home, this may indicate that their family is unable to manage their illness, and, without additional support, the child’s health may deteriorate, and they may re-enter care again. For those who are young adults, aged 16 or above, our results echo previous research that has argued for a more holistic, gradual and flexible approach to the transition into independent life for young care leavers.^30^

### Strengths and limitations

The strengths of our work include high-quality population-wide longitudinal data on hospitalisations and childhood social care. For the first time in the UK, this allows us to explore differences in hospitalisations for the three most common chronic conditions leading to hospital admissions between care experienced and general population CYP. Importantly, we also distinguish between whether hospitalisations occur before, during or after care and estimate the effect of each of these periods on hospitalisation hazards.

The most significant limitation of our work is not having data on doctor’s diagnosis, and we rely on hospitalisation and prescription records to estimate the prevalence of the three conditions. We are likely to correctly identify children admitted to hospital as having one of the three conditions but not all children with chronic illnesses are hospitalised. We have attempted to capture those who are never hospitalised by including prescriptions for asthma and diabetes but are still likely to miss some children with these conditions. This is most likely to affect our estimation of asthma prevalence among the oldest members of our cohort who may have recovered from the condition before 2009 and have never been hospitalised or had a prescription since 2009. Our estimation of prevalence is improved by having a long time series as this increases the likelihood of hospitalisations and receiving a prescription.

Estimating epilepsy prevalence is the most difficult. We have not included epilepsy prescriptions in prevalence estimates as research has shown that these medications are increasingly used to treat other conditions. Therefore, our prevalence only relies on those CYP who have been in hospital. However, epilepsy hospitalisations themselves may be misdiagnosed, for example mistakenly include PNES. Therefore, most caution is needed when interpreting these results.

## Conclusions

Our work is the first in the UK to show that while differences in the prevalence of physical ill health between care experienced and general population CYP might not be present at childhood, differences in hospitalisation rates are evident at early ages and even before children enter care. Hospitalisation rates are higher outside care episodes, such as before entering and after leaving care. High hospitalisations prior to care indicate that, for many families, managing childhood chronic health conditions adequately is a challenge. Inadequate management of chronic conditions in childhood can lead to significantly higher levels of ill health in adulthood and better policies and practices need to be implemented to help families and CYP successfully manage these illnesses. These policies may include practical help in accessing services but also more accepting non-judgemental attitudes on the part of healthcare professionals. The results also suggest that leaving care can be a difficult period of rapid change and young people starting independent life will need more holistic (emotional, social, practical and financial) support to take care of their health.

## Supplementary material

10.1136/bmjpo-2024-002705 online supplemental file 1

10.1136/bmjpo-2024-002705 online supplemental file 2

## Data Availability

Data may be obtained from a third party and are not publicly available.
