# Correction for ‘Hospitalisations for chronic conditions among care experienced and general population children and young people: evidence from the Children’s Health in Care in Scotland (CHiCS) cohort study, 1990–2016’

**DOI:** 10.1136/bmjpo-2024-002705corr1

**Published:** 2025-04-17

 Allik M, Gedeon E, Henderson M, *et al*. Hospitalisations for chronic conditions among care experienced and general population children and young people: evidence from the Children’s Health in Care in Scotland (CHiCS) cohort study, 1990–2016. *BMJ Paediatrics Open* 2024;8:e002705. doi:10.1136/bmjpo-2024–0 02 705

This article has been corrected since it was published online. The text on page 2 under Data and Methods that read:

‘Our data include age (in months) for each hospitalisation and, for care experienced children, also the age (in months) at which they entered care, changed care placement (such as between different types of care) or left care.’

Has now been replaced by (changes highlighted):

‘Our data include age (in months) for each hospitalisation and, for care experienced children, also the age (in months) **at which their care episode started**, changed care placement (such as between different types of care) or left care. This means that we can place each hospitalisation to a specific time point in a child’s life course and journey through care, that is, we know which hospitalisations occurred before, during or after leaving social care.

There are, however, some limitations to this. While most children who enter care only have one episode of care, that is, they enter care, are in care and then leave care, some experience multiple episodes. Our data does not capture episodes of care which ended before April first 2008, meaning that a small proportion of early episodes of care are excluded. See Supplement 2 for further details.

**Also,** if a child leaves care before the age of 16, it is possible for them to re-enter care. In our data, the majority of children who left social care were aged 16 or older at the end of our study, meaning that they will not re-enter care. Others may have re-entered care after the end of the follow-up period. This means that, in our data, the category of children who have left care is heterogeneous, though most will be young adults who have permanently left care.’

In addition, supplement 2 has been added to the article.

In addition, there was a typographical error in table 1. Line 2 “N Hospitalisations/Mean” previously red for asthma and diabetes

**Table:** 

	Asthma				Diabetes			
	GPC		CEC		GPC		CEC	
	N	%	N	%	N	%	N	%
N Hospitalisations/Mean	15 695	0.17	478	0.21	6150	1.1	311	2.2

But should read

**Table:** 

	Asthma				Diabetes			
	GPC		CEC		GPC		CEC	
	N	%	N	%	N	%	N	%
N Hospitalisations/Mean	33.030	0.35	1055	0.47	11 167	2.0	628	4.5

