# Supplementary material for: Hospitalisations for chronic conditions among care experienced and general population children and young people: evidence from the Children’s Health in Care in Scotland (CHiCS) cohort study, 1990–2016
Source: BMJ Paediatr Open. 2024 Oct 2;8(1):e002705. doi: 10.1136/bmjpo-2024-002705 (PMC11448190; doi:10.1136/bmjpo-2024-002705)
Supplement: online supplemental file 1 [file bmjpo-8-1-s001.pdf]

Supplement

Table S - 1. Distribution of explanatory variables by chronic condition and cohort, birth records data. GPC – General population children; CEC – Care experienced children.

	Asthma				Diabetes				Epilepsy			
	GPC		CEC		GPC		CEC		GPC		CEC	
	N	%	N	%	N	%	N	%	N	%	N	%
Children with BR	86,808	91.7	2,072	92.4	5,015	91.2	126	88.7	2,939	93.2	153	95.6
Mother's age at birth (years)												
Median/IQR (years)	28	8	23	8	28	8	23	9	28	9	24	8
Mean/SD (years)	28.0	5.7	24.4	5.8	28.1	5.5	24.3	6.4	27.6	5.9	25.4	6.2
Missing*	86		2		6		0		0		0	
Parent SES**												
0: Student/Unemployed/Not available	6,329	7.3	599	28.9	305	6.1	33	26.2	273	9.3	37	24.2
1: Employee	62,435	71.9	1,346	65.0	3,682	73.4			2,101	71.5	79	51.6
2: Manager/Supervisor	10,803	12.4	66	3.2	602	12.0	93	73.8	367	12.5		
4: Self-employed (with/without employees)	7,241	8.3	61	2.9	426	8.5			197	6.7	14	9.2
N complete observations	86,728		1,870		5,009		107		2,938		130	

*Number represents those with BR present, but mother's age not filled in.

**For children born prior to 1996, parental employment status was based on one occupation only; father's occupation if married, otherwise mother's occupation. For births 1996 to 2004, both parent's occupation was recorded for all births registered by married couples or for births that were jointly registered by unmarried couples. The data is harmonised across 1990 to 2004 such that we take father's occupation between 1996 to 2004, if available, otherwise mother's occupation.

Table S - 2. Age standardised and crude rates of asthma, diabetes, and epilepsy in the CHiCS study, Scotland, and England

**Condition**	**Standardised rate***		**Crude rate****		
	**Rate**	**95% CI**	**CHiCS, 1990-2016**	**Scotland, range 2011-2016**	**England, range 2011-2016**
Asthma	248.4	244.9-252.0	298	200-219	186-211
Diabetes	137.2	133.7-140.7	85	85-97	53-59
Epilepsy	81.5	79.3-83.7	88	83-106	70-74

*Age-standardised rates for hospitalisations per 100,000 person-years for both cohorts, ages [0-25) using 2013 ESP.

**Crude rates per 100,000 population. For CHiCS study the rate is across the noted years for those under 18 years of age. Scottish rates are from Public Health Scotland for children aged 18 and under. English rates are for those aged 19 and under, authors' calculations based on NHS Digital data. For England, diabetes hospitalisations only include ICD-10 code E10 (type 1).

Table S - 3. Poisson models RR and 95% CI for the number of hospitalisations for children with birth records (including mothers age and parent employment at birth).

	Asthma			Diabetes			Epilepsy		
		95% CI			95% CI			95% CI	
**Variable**	**RR**	**Low**	**High**	**RR**	**Low**	**High**	**RR**	**Low**	**High**
Intercept	0.01	0.01	0.02	0.12	0.11	0.13	0.14	0.13	0.15
Ref: Female GP									
Male GP	1.29	1.26	1.32	0.97	0.93	1.01	0.89	0.85	0.93
Care experienced female	0.95	0.85	1.06	1.38	1.21	1.57	1.24	1.06	1.44
Care experienced Male	1.58	1.45	1.71	2.30	2.00	2.63	1.30	1.16	1.44
Deprivation (ref 1 - Low):									
2	1.12	1.08	1.17	1.03	0.96	1.10	1.03	0.96	1.11
3	1.07	1.02	1.11	1.03	0.96	1.10	1.10	1.03	1.18
4	1.28	1.23	1.34	1.09	1.02	1.16	0.97	0.91	1.04
5- High	1.36	1.31	1.42	1.19	1.12	1.27	0.90	0.84	0.96
Rural (ref Urban)	0.94	0.91	0.96	0.85	0.82	0.89	0.97	0.92	1.01
Co-morbid	1.41	1.36	1.47	0.97	0.93	1.02	1.31	1.26	1.37
Disabled	1.14	1.08	1.21	0.85	0.79	0.92	2.20	2.11	2.30
Year of birth	1.00	1.00	1.00	1.02	1.01	1.02	1.07	1.06	1.07
Mother's age at birth	0.97	0.97	0.98	0.98	0.98	0.98	1.00	0.99	1.00
Parent's SES (ref Student/Unemployed/Not available)									
1: Employee	0.96	0.92	0.99	1.18	1.10	1.26	1.00	0.93	1.07
2: Manager/Supervisor	1.01	0.96	1.06	0.87	0.81	0.93	0.88	0.82	0.94
3: Self-employed	0.96	0.91	1.02	0.96	0.89	1.03	0.98	0.90	1.06
N Children			88,598			5,116			3,068

Table S - 4. Event history model HR and 95% CI for children with birth records (including mothers age and parent employment at birth). Strata include co-morbidities, disabilities, and birth cohort.

	Asthma			Diabetes			Epilepsy		
		95% CI			95% CI			95% CI	
**Variable**	**HR**	**Low**	**High**	**HR**	**Low**	**High**	**HR**	**Low**	**High**
Reference: never in care									
Before care	1.05	0.90	1.23	1.61	1.06	2.44	1.65	1.16	2.36
In care	1.23	0.74	2.03	1.14	0.76	1.73	0.99	0.68	1.44
After care	1.21	0.79	1.84	2.25	1.42	3.57	1.24	0.81	1.90
Male	1.30	1.22	1.38	1.00	0.89	1.12	0.90	0.80	1.02
Deprivation (ref 1 - Low):									
2	1.12	1.02	1.24	1.03	0.88	1.20	1.04	0.85	1.27
3	1.06	0.96	1.17	1.03	0.86	1.22	1.11	0.89	1.39
4	1.28	1.16	1.42	1.09	0.92	1.29	0.97	0.80	1.18
5- High	1.36	1.24	1.50	1.20	0.99	1.45	0.91	0.75	1.10
Rural (ref Urban)	0.94	0.88	1.00	0.85	0.76	0.95	0.95	0.84	1.09
Mother's age at birth	0.97	0.97	0.98	0.98	0.97	0.99	1.00	0.99	1.01
Parent's SES (ref Student/Unemployed/Not available)									
1: Employee	0.96	0.86	1.08	0.84	0.63	1.13	1.00	0.80	1.24
2: Manager/Supervisor	1.02	0.89	1.17	0.74	0.55	0.99	0.87	0.65	1.17
3: Self-employed	0.97	0.84	1.13	0.82	0.59	1.14	0.97	0.74	1.29
N hospitalisations			32,221			10,835			9,606
N children			88,598			5,116			3,068

Table S - 5a. Event history models for males

Males	Asthma			Diabetes			Epilepsy		
		95% CI			95% CI			95% CI	
**Variable**	**exp(b)**	**Low**	**High**	**exp(b)**	**Low**	**High**	**exp(b)**	**Low**	**High**
Reference: never in care									
Before care	1.15	0.98	1.36	2.87	1.73	4.76	1.87	1.21	2.89
In care	1.71	0.88	3.31	1.67	1.01	2.78	0.86	0.58	1.26
After care	1.61	0.92	2.79	2.85	1.62	5.03	1.80	1.05	3.09
Deprivation (ref 1 - Low):									
2	1.14	1.02	1.27	0.96	0.81	1.13	1.03	0.78	1.35
3	1.18	1.05	1.31	0.94	0.79	1.13	0.96	0.76	1.22
4	1.35	1.22	1.48	1.37	1.11	1.69	0.99	0.78	1.26
5- High	1.46	1.32	1.61	1.47	1.18	1.83	1.02	0.79	1.33
Rural (ref Urban)	0.93	0.86	1.00	0.97	0.84	1.11	0.85	0.73	0.98
N hospitalisations		20,375			5,271			5,491	
N children		52,391			2,584			1,761	

Table S - 5b. Event history models for females

Females	Asthma			Diabetes			Epilepsy		
		95% CI			95% CI			95% CI	
**Variable**	**exp(b)**	**Low**	**High**	**exp(b)**	**Low**	**High**	**exp(b)**	**Low**	**High**
Reference: never in care									
Before care	1.07	0.78	1.45	1.29	0.77	2.17	1.37	0.82	2.30
In care	0.79	0.56	1.12	1.05	0.63	1.73	1.26	0.66	2.42
After care	1.15	0.64	2.07	2.02	1.06	3.85	0.73	0.34	1.56
Deprivation (ref 1 - Low):									
2	1.12	0.94	1.33	1.08	0.86	1.37	1.05	0.81	1.37
3	1.03	0.86	1.22	1.25	0.97	1.62	1.41	1.00	1.98
4	1.41	1.18	1.69	1.18	0.94	1.48	1.07	0.80	1.41
5- High	1.65	1.40	1.95	1.27	0.98	1.65	0.92	0.72	1.16
Rural (ref Urban)	0.94	0.84	1.04	0.81	0.69	0.95	1.04	0.85	1.27
N hospitalisations		13,608			6,405			4,727	
N children		44,319			3,036			1,525	

Table S – 6. Event history models with care placement type

	Asthma			Diabetes			Epilepsy		
		95% CI			95% CI			95% CI	
**Variable**	**exp(b)**	**Low**	**High**	**exp(b)**	**Low**	**High**	**exp(b)**	**Low**	**High**
Reference - never in care									
Before care	1.11	0.95	1.29	1.92	1.31	2.81	1.73	1.23	2.44
At home	1.43	0.91	2.26	1.49	0.92	2.40	1.33	0.75	2.37
Kinship	1.02	0.63	1.65	1.15	0.45	2.95	0.87	0.36	2.11
Fostering	1.47	0.49	4.39	1.04	0.58	1.86	0.79	0.50	1.24
Residential	0.93	0.39	2.17	1.88	0.67	5.29	1.37	0.44	4.31
After care	1.36	0.91	2.04	2.42	1.57	3.74	1.39	0.89	2.19
Male	1.28	1.20	1.36	1.02	0.91	1.14	0.93	0.82	1.04
Deprivation (ref 1 - Low):									
2	1.13	1.03	1.24	1.02	0.88	1.18	1.02	0.84	1.24
3	1.12	1.02	1.23	1.09	0.92	1.29	1.14	0.93	1.41
4	1.37	1.25	1.50	1.25	1.07	1.47	1.01	0.84	1.21
5- High	1.53	1.40	1.68	1.35	1.14	1.61	0.98	0.81	1.17
Rural (ref Urban)	0.93	0.87	0.99	0.88	0.79	0.98	0.94	0.83	1.07
N hospitalisations			33,983			11,676			10,181
N children			96,710			5,620			3,286

Table S - 7 Event history models with interactions between sex and age group (age included in the models as strata)

	Asthma			Diabetes			Epilepsy		
		95% CI			95% CI			95% CI	
**Variable**	**exp(b)**	**Low**	**High**	**exp(b)**	**Low**	**High**	**exp(b)**	**Low**	**High**
Reference - never in care									
Before care	1.11	0.96	1.30	1.90	1.29	2.78	1.72	1.22	2.43
In care	1.29	0.79	2.10	1.31	0.91	1.88	0.97	0.68	1.39
After care	1.35	0.90	2.02	2.39	1.55	3.69	1.40	0.89	2.19
Male aged <12	1.55	1.47	1.63	1.11	1.02	1.22	0.94	0.81	1.08
Male aged ≥12	0.63	0.54	0.74	0.97	0.83	1.14	0.90	0.75	1.08
Deprivation (ref 1 - Low):									
2	1.13	1.03	1.24	1.02	0.88	1.18	1.03	0.85	1.25
3	1.12	1.02	1.23	1.09	0.92	1.29	1.14	0.93	1.41
4	1.37	1.25	1.51	1.25	1.07	1.47	1.01	0.84	1.21
5- High	1.53	1.40	1.68	1.35	1.13	1.61	0.98	0.81	1.17
Rural (ref Urban)	0.93	0.87	0.99	0.88	0.79	0.98	0.95	0.83	1.07
N hospitalisations			33,983			11,676			10,181
N children			96,710			5,620			3,286
