# Supplementary material for: Hospitalisations for chronic conditions among care experienced and general population children and young people: evidence from the Children’s Health in Care in Scotland (CHiCS) cohort study, 1990–2016
Source: BMJ Paediatr Open. 2024 Oct 2;8(1):e002705. doi: 10.1136/bmjpo-2024-002705 (PMC11448190; doi:10.1136/bmjpo-2024-002705)
Supplement: online supplemental file 2 [file bmjpo-8-1-s002.docx]

## Supplement 2 – Addressing potential missing episodes of care

The formal national data collection of childhood social care statistics in Scotland started in 2008 and information on care histories prior to 1 April 2008 is included where the child has continuously been in care until a date on or after 1 April 2008. This means that episodes of care that started and ended before April 1, 2008, are excluded from the data. This issue is visualised in Soraghan and Porter 2024 (Figure 1, p16)

Most children who enter care only have one episode of care, that is, they enter care, are in care (during which their placement may change) and then leave care. Therefore, only a minority of children in our data would have had episodes of care prior to 2008 that are absent from our data. Different sources suggest that about 16%-18% of children in care have had more than 1 episode (e.g. Soraghan and Porter 2024 p31 and MacIntyre, Kellock, Alexis and Waddell 2024 p5). Because the population affected by this is relatively small, it does not have substantial implications for the presented work. However, to show that accounting for this exclusion does not affect our conclusions, we included further analysis in this supplement to account for this.

Table S.2 - 1 shows the number and proportion of children in care by the number of episodes of care (between August 1^st^ 2009 - July 31^st^ 2017) and birth cohort in our data. For the younger cohorts, for whom data coverage is more complete, the proportion of children with 2 or more episodes is higher. There is no variation in the proportion of children by the number of episodes and birth cohort if we only look at those with asthma, diabetes or epilepsy.

Table S.2 - 1 The number and proportion of care experienced children by the number of episodes and birth cohort.

|  | **Number of children** | | | |  | **Proportion of children** | | | |
| --- | --- | --- | --- | --- | --- | --- | --- | --- | --- |
| **Birth cohort** | **1 Episode** | **2 or more*** |  | **Total** |  | **1 Episode** | **2 or more*** |  | **Total** |
| **[1990,1996)** | 4,712 | 117 |  | 4,829 |  | 97.6 | 2.4 |  | 100 |
| **[1996,2000)** | 4,024 | 364 |  | 4,388 |  | 91.7 | 8.3 |  | 100 |
| **[2000,2004]** | 4,092 | 521 |  | 4,613 |  | 88.7 | 11.3 |  | 100 |
|  |  |  |  |  |  |  |  |  |  |
| **Total** | 12,828 | 1,002 |  | 13,830 |  | 92.8 | 7.2 |  | 100 |
| *The proportion of children with 3 or 4 episodes was extremely small and we are unable to provide separate counts for these due to statistical disclosure control. | | | | | | | | | |

The mean length of time between episodes in our data was 18 months (and 12-19 months if only considering those with asthma, diabetes or epilepsy) and the median 10 months (11 months for those with asthma, diabetes and epilepsy).

Based on the mean and median lengths we have provided supplemental analysis which left-truncates the “before care” period to 12 months before the first know entry to care. This should substantially reduce the odds of any child being in care during the “before care” period. Sensitivity analysis was conducted where the “before care” period was truncated to 6 months before the first known entry to care. The truncation resulted in removing some hospitalisations and reducing person-years from the “before care” period, but the number of children included in the analysis remains the same. Table S.2 - 2 shows the proportion of hospitalisations removed and remaining for each of the conditions and the proportion of person-years reduced. The proportion of hospitalisations remaining is higher for all conditions compared to the proportion of person-years, especially for diabetes and epilepsy. Further descriptive analysis (histograms) showed that the number of hospitalisations increased in the 1-2 years before entering care compared to more than 2 years before care. (Histograms are not provided due to statistical disclosure control as they show small extreme frequencies.)

Table S.2 - 2 The proportion of hospitalisations and person-years removed and remaining in models due to 12-month truncation.

|  | **Removed** | **Remaining** |
| --- | --- | --- |
| **N asthma hospitalisations** | 87.5 | 12.5 |
| **N diabetes hospitalisations** | 70.1 | 29.9 |
| **N epilepsy hospitalisations** | 81.3 | 18.7 |
|  |  |  |
| **PY for those in care** | 89.0 | 11.0 |

Table S.2 - 3 below presents the results from repeated events event history models where the “Before care” period only includes 12 months before the first known episode of care. The results of these models are similar to those presented in the main article Table 3. The most notable differences are the higher hazard ratios (HR) for the before care period for all three conditions. For asthma, the 95% confidence intervals (CI) for the HR now narrowly exclude 1 (HR=1.50, CI=1.05-2.14) while in the main article they narrowly included 1 (HR=1.11, CI=0.95-1.29). For diabetes and epilepsy, the CI excluded 1 in the main article table also, but the effect size is larger in the truncated models presented here. There are only marginal changes to the HR and CI of other variables.

Results for the same models using the 6-month truncation are very similar to those presented in Table S.2 - 3. These models include all the same children, but only the events and person years 6 months leading up to the first known episode of care. For asthma, these models had a similar HR, but the CI were wider and included 1. For diabetes the HR were also the same as in Table S.2 – 3, the CI were wider but did not include 1. For epilepsy the HR was slightly smaller and CI wider but did not include 1. There was no change in the HR or CI of other variables.

### References

Soraghan, J. & Porter, R. B. (2024) Growing Up in Kinship Care, 8 Oct 2024, 82 p.

<https://pure.strath.ac.uk/ws/portalfiles/portal/242110939/Soraghan-Porter-SCADR-2024-Growing-Up-in-Kinship-Care.pdf>

MacIntyre, C.; Kellock, C.; Alexis, T. and Waddell, R. (2024) Looked after children in Scotland - longitudinal data user guide <https://www.adruk.org/data-access/flagship-datasets/looked-after-children-longitudinal-dataset/>

Table S.2 - 3 Hazard ratios (HR) and 95% CI for repeated events event history models for hospitalisations for asthma, diabetes, and epilepsy. Before care period left-truncated to only include 12 months before first known episode of care. Strata include co-morbidities, disabilities, and birth cohort.

|  |  | **Asthma** |  |  |  | **Diabetes** |  |  |  | **Epilepsy** |  |
| --- | --- | --- | --- | --- | --- | --- | --- | --- | --- | --- | --- |
|  |  | **95% CI** | |  |  | **95% CI** | |  |  | **95% CI** | |
| **Variable** | **HR** | **Low** | **High** |  | **HR** | **Low** | **High** |  | **HR** | **Low** | **High** |
| Reference: never in care |  |  |  |  |  |  |  |  |  |  |  |
| Before care | 1.50 | 1.05 | 2.14 |  | 3.16 | 1.99 | 5.02 |  | 3.00 | 1.63 | 5.52 |
| In care | 1.29 | 0.79 | 2.10 |  | 1.31 | 0.91 | 1.88 |  | 0.98 | 0.68 | 1.41 |
| After care | 1.36 | 0.91 | 2.04 |  | 2.40 | 1.55 | 3.70 |  | 1.40 | 0.89 | 2.20 |
|  |  |  |  |  |  |  |  |  |  |  |  |
| Male | 1.27 | 1.20 | 1.36 |  | 1.01 | 0.91 | 1.14 |  | 0.91 | 0.81 | 1.03 |
|  |  |  |  |  |  |  |  |  |  |  |  |
| Deprivation (ref 1 - Low): |  |  |  |  |  |  |  |  |  |  |  |
| 2 | 1.13 | 1.03 | 1.24 |  | 1.02 | 0.88 | 1.18 |  | 1.03 | 0.85 | 1.25 |
| 3 | 1.11 | 1.01 | 1.22 |  | 1.10 | 0.93 | 1.30 |  | 1.13 | 0.91 | 1.40 |
| 4 | 1.37 | 1.25 | 1.50 |  | 1.25 | 1.07 | 1.47 |  | 1.01 | 0.84 | 1.22 |
| 5- High | 1.54 | 1.41 | 1.68 |  | 1.36 | 1.14 | 1.62 |  | 0.96 | 0.80 | 1.16 |
|  |  |  |  |  |  |  |  |  |  |  |  |
| Rural (ref Urban) | 0.93 | 0.88 | 0.99 |  | 0.88 | 0.79 | 0.98 |  | 0.95 | 0.83 | 1.08 |
|  |  |  |  |  |  |  |  |  |  |  |  |
| N events (all hospitalisations) |  |  | 33,491 |  |  |  | 11,580 |  |  |  | 9,922 |
| N children |  |  | 96,710 |  |  |  | 5,620 |  |  |  | 3,286 |
