# Supplementary material for: Hospitalisations for chronic conditions among care experienced and general population children and young people: evidence from the Children’s Health in Care in Scotland (CHiCS) cohort study, 1990–2016
Source: BMJ Paediatr Open. 2024 Oct 2;8(1):e002705. doi: 10.1136/bmjpo-2024-002705 (PMC11448190; doi:10.1136/bmjpo-2024-002705)
Supplement: Supplementary file 3 [file bmjpo-2024-002705.reviewer_comments.pdf]

PEER REVIEW HISTORY

BMJ Paediatrics Open publishes all reviews undertaken for accepted manuscripts. Reviewers are asked to complete a checklist review form and are provided with free text boxes to elaborate on their assessment. These free text comments are reproduced below.

ARTICLE DETAILS

**TITLE (PROVISIONAL)**	Hospitalisations for chronic conditions among care-experienced and general population children and young people: evidence from the Children's Health in Care in Scotland (CHiCS) cohort study, 1990-2016
**AUTHORS**	Allik, Mirjam; Gedeon, Edit; Henderson, Marion; Leyland, Alastair

VERSION 1 - REVIEW

**REVIEWER NAME**	***Doug Simkiss***
**REVIEWER AFFILIATION**	University of Warwick
**REVIEWER CONFLICT OF INTEREST**	
**DATE REVIEW RETURNED**	16-May-2024

**GENERAL COMMENTS**	This is an interesting paper with novel and important findings on children in care / care leavers with asthma, diabetes and epilepsy as sentinel long term conditions. I enjoyed reading the paper.<br><br>There are some issues that I think you should consider before publication.<br><br>1. What is the hypothesis driving the research? I could not find an hypothesis that you were testing. I was left wondered whether you were expecting to find fewer care experienced children being admitted to hospital because there are access issues for them or<br>2. Whether you were expecting to find more care experienced children admitted than their peers because they have poorer control of their long term conditions.<br><br>As I read the paper I appreciated that you segment the children's journey into pre-care, during care and after a period of care so the hypothesis because more complex than I set out above, but I was expecting an hypothesis to test nonetheless.<br><br>Secondly, I note the more common occurrence of epilepsy in this group. One contentious reason for this increase that you do not discuss is the possibility of psychogenic non-epileptic seizures. These are events that appear to be epileptic seizures but, in fact, do not represent the manifestation of abnormal excessive synchronous cortical activity, which defines epileptic seizures. They are not a variation of epilepsy but are of psychiatric origin. In my clinical practice I have seen these in children in care and I think you should investigate this a little further to see if you want to mention it as a possible cause (or not as I understand it is contentious)<br><br>Thirdly, I just want to you to confirm that you have addressed the possibility of identifying the foster carer or children's home address in creating your socio-economic status variable. I have seen

	researchers pick up the carers address rather than the birth families and saying that you are aware of this trap and have mitigated against it would be useful.<br><br>Fourthly, you have discussed ACE's at some length. There are papers that directly link ACE's to children in care - The needs of looked after children from an adverse childhood experience perspective (https://doi.org/10.1016/j.paed.2018.11.005) is one.<br><br>Fifthly, you make sensible recommendations for family support, but you don't seem to have considered the likely possibility that for some of these children it is the hospital admission itself that precipitates the care episode. It is not uncommon in clinical practice for the poor control of a long term condition requiring admission to hospital to become an issue of neglect, resulting in a child protection plan and sometimes an admission to foster care direct from hospital. Are you able to date the admission to care in relation to hospital admission?<br><br>Finally, I was interested if you know the age of children you are describing as 'after care'? That phrase could be interpreted as an adult care leaver who has been in care through adolescence, but some children get returned home after an episode of care while still children and I was not sure if those children are included in the after care grouping. The reason for being explicit is that the former group need help taking control of their long term condition management while the latter may well still be necessarily dependent on the adults in their life.

**REVIEWER NAME**	***Madeleine Powell***
**REVIEWER AFFILIATION**	University of New South Wales, School of Population Health
**REVIEWER CONFLICT OF INTEREST**	
**DATE REVIEW RETURNED**	26-May-2024

**GENERAL COMMENTS**	Madeleine Powell<br><br>This study utilises data from the CHICS linked administrative data cohort. It included a population of 13,830 children who received foster care and a general population of 649,771 children who did not receive care during the study period, who were in school in 2009 in Scotland (born from 1990 to 2004). The study ascertains hospitalisation, prescription, and care records for the children from birth up to July 2016, with differing follow up time for different birth cohorts. Outcomes included the prevalence of chronic diseases Type 1 diabetes, epilepsy, and asthma among both the foster care and general populations. The authors also report the hazard ratio of hospitalisations among children in care and the general population, stratified by chronic condition.<br>This study has valuable descriptive data on the prevalence of chronic conditions among these populations, and the incidence of hospitalisations among these groups. However, currently the way the analysis has been conducted indicates that there is some confusion about the effects that are being estimated. For example, in the time-varying analysis, described on line 4 of page 5, the incidence of hospitalisations, stratified by chronic condition, among children who have never been in care is compared to children who will one day enter care. So, this is measuring the effect of care

placement before the exposure (foster care) has occurred. While the authors do state that increased hazard ratio seen here is due to confounding from pre-existing factors within the family etc., as it cannot be caused be entering care, this analysis does not meet the rule of temporality, i.e. that the exposure occurs before the outcome. This result also has limited policy and practice relevance as you cannot intervene based on an exposure that has not yet happened.

Another analysis choice that makes the results difficult to interpret is comparing males who experienced care to females who did not experience care in the general linear model reported in figure 1 and table 2. If sex plays a role in the incidence of hospitalisations and care entry, thus confounding the effect, it would be preferable to handle confounding by stratifying the results be sex, so comparing males in care to males in general population, and females in care to females in the general population.

Recommendations for analysis and reporting of results:

• I recommend that the authors follow the RECORD guidelines for reporting observational studies, and particularly provide flow diagrams that illustrate which populations are included in the different analyses.
• I recommend reporting only the results of the current study in your results section and reporting comparisons to other studies in the discussion (e.g., lines 3-7 and 11-14 on page 22).
• When reporting results, I recommend reporting the prevalence of the outcomes in each population group and the risk difference between groups, as well as the relative risk. For example, while the authors report that relative risk of epilepsy among the foster care group compared to the general population group is the highest (see line 59-60 pg5, and line 10 pg8), the risk difference between the two populations is 0.7%. Whilst this equates to a relative risk of 2.4, in absolute terms it means that an additional 7 children out of every 1000 children in the care population have epilepsy compared to every 1000 children of the general population. For asthma whilst the relative risk is not large, the risk difference between the groups is greater than epilepsy (1.6%) which equates to an additional 16 children out of every 1000 having asthma, compared to every 1000 children in the general population. So, whilst the relative risk makes the difference between groups for epilepsy seem the most substantial, the absolute risk difference and prevalence estimates show that in terms of actual numbers of children, the increase in asthma in the care group has potentially more impact on service delivery.
• I recommend reporting the results of sensitivity analyses in the supplementary material rather than stating "(not shown)" (Line 29 pg 6).
• Table 1: I would recommend:
o reporting the descriptive variables unstratified by care group and unstratified by chronic condition. This would provide a reference for the values within these groups, e.g., providing an all-children column, and all children in care and general population columns.
o providing the denominator for the prevalence estimates, for example providing the total number of children in care and general populations.
o keeping the decimal places consistent for the hospitalisation means reported (e.g., mean hospitalisations for asthma reported to 1 decimal place).

	o For deprivation in the diabetes CEC column, what row do the 17 and 12.0 values correspond to, 1-Low or 2?<br><br>26-May-2024

VERSION 1 – AUTHOR RESPONSE

Reviewer: 1

Prof. Doug Simkiss, University of Warwick, Aston University

Comments to the Author

1. What is the hypothesis driving the research? I could not find a hypothesis that you were testing. I was left wondered whether you were expecting to find fewer care experienced children being admitted to hospital because there are access issues for them or 2. Whether you were expecting to find more care experienced children admitted than their peers because they have poorer control of their long term conditions. As I read the paper I appreciated that you segment the children's journey into pre-care, during care and after a period of care so the hypothesis because more complex than I set out above, but I was expecting an hypothesis to test nonetheless.

We have now included the hypothesis at the end of the introduction. There is not much evidence to guide our hypothesis regarding the before, during and after care periods, and therefore we simply assume that social care is doing what it is supped to and supports children's health and wellbeing.

Secondly, I note the more common occurrence of epilepsy in this group. One contentious reason for this increase that you do not discuss is the possibility of psychogenic non-epileptic seizures. These are events that appear to be epileptic seizures but, in fact, do not represent the manifestation of abnormal excessive synchronous cortical activity, which defines epileptic seizures. They are not a variation of epilepsy but are of psychiatric origin. In my clinical practice I have seen these in children in care and I think you should investigate this a little further to see if you want to mention it as a possible cause (or not as I understand it is contentious)

Thank you for raising this, especially as we lack a clinical perspective. It is a difficult issue to address. From or reading of the evidence, the psychogenic non-epileptic seizures (PNES) are coded in ICD-10 as F44 Dissociative disorders, e.g. F44.5 Dissociative convulsions – "Dissociative convulsions may mimic epileptic seizures very closely in terms of movements, but tongue-biting, bruising due to falling, and incontinence of urine are rare, and consciousness is maintained or replaced by a state of stupor or trance." Therefore, if correctly diagnosed, we should be excluding these cases and we have noted this now in the methods section where we provide our definitions. However, misdiagnosis is, of course, possible. Unfortunately, we do not know who have/have not been misdiagnosed and we have made that clear in the text.

Thirdly, I just want to you to confirm that you have addressed the possibility of identifying the foster carer or children's home address in creating your socio-economic status variable. I have seen

researchers pick up the carers address rather than the birth families and saying that you are aware of this trap and have mitigated against it would be useful.

Yes, these are two quite different things and in our cohort profile paper referenced in the manuscript we briefly looked at this issue (see Table 3 in Allik et al 2020 http://dx.doi.org/10.1136/bmjopen-2021-054664)

Here, we used the home address included in the birth registrations whenever possible to derive the SES variable (deprivation). As it is the birth registrations data, there should not be any possibility that this is the carers address. Birth registrations are available for those born in Scotland (88% as noted in paper). This excludes children born in England, Norther Ireland, Wales or elsewhere in the world. For those children we use the SES from the education data (Pupil Census) and that could be the carer's address. Unfortunately, we have no way of knowing which that is. Therefore, for a small proportion of children, this might be the carer's address. This has now been highlighted in the text also.

Fourthly, you have discussed ACE's at some length. There are papers that directly link ACE's to children in care - The needs of looked after children from an adverse childhood experience perspective (https://doi.org/10.1016/j.paed.2018.11.005) is one.

Thank you, we have included a reference that directly links social care to very high levels of ACEs in the introduction.

Fifthly, you make sensible recommendations for family support, but you don't seem to have considered the likely possibility that for some of these children it is the hospital admission itself that precipitates the care episode. It is not uncommon in clinical practice for the poor control of a long term condition requiring admission to hospital to become an issue of neglect, resulting in a child protection plan and sometimes an admission to foster care direct from hospital. Are you able to date the admission to care in relation to hospital admission?

Yes, we are able to date the admission to care in relation to hospital admissions! We do believe that hospital admissions and poor management of illness may contribute to a child entering care. Our data sets include age (in moths) for each hospitalisation and for the start and end of each care placement. We use these data in our event history models to link each of the hospitalisations to the periods of before, during and after care. We have updated the methods section to note this more clearly. Our recommendation of family support reflects just that – we recommend this support to all children with chronic illnesses to help families cope and reduce the chances of the child entering care in the first place.

Finally, I was interested if you know the age of children you are describing as 'after care'? That phrase could be interpreted as an adult care leaver who has been in care through adolescence, but some children get returned home after an episode of care while still children and I was not sure if those children are included in the after care grouping. The reason for being explicit is that the former group need help taking control of their long term condition management while the latter may well still be necessarily dependent on the adults in their life.

That is correct, this is a heterogenous group. The majority of those in “after care” will be care leavers who cannot re-enter care. But there are those who are under the age of 16 and can re-enter care. We have clarified that point now in the methods section. Our recommendation in the article is that the adult care leavers, who are only 16-18, will still need help in taking control of their conditions and we have made that explicit now also.

Reviewer: 2

Dr. Madeleine Powell, University of New South Wales

currently the way the analysis has been conducted indicates that there is some confusion about the effects that are being estimated. For example, in the time-varying analysis, described on line 4 of page 5, the incidence of hospitalisations, stratified by chronic condition, among children who have never been in care is compared to children who will one day enter care. So, this is measuring the effect of care placement before the exposure (foster care) has occurred. While the authors do state that increased hazard ratio seen here is due to confounding from pre-existing factors within the family etc., as it cannot be caused be entering care, this analysis does not meet the rule of temporality, i.e. that the exposure occurs before the outcome. This result also has limited policy and practice relevance as you cannot intervene based on an exposure that has not yet happened.

We disagree with the statement that we are “measuring the effect of care placement before the exposure (foster care) has occurred” or that our analysis “does not meet the rule of temporality, i.e. that the exposure occurs before the outcome”. For those who experience care, the time we observe them in the study has been split into three broad groups: 1) time before care, 2) time in care and 3) time after care. The effects for time before care is estimated separately from the effects of time spent in care and also separately from the effects of time after leaving care. The effect of actually being in care is only estimated for the time actually in care. We extended our methos section to clarify this point more in the methods section.

Another analysis choice that makes the results difficult to interpret is comparing males who experienced care to females who did not experience care in the general linear model reported in figure 1 and table 2. If sex plays a role in the incidence of hospitalisations and care entry, thus confounding the effect, it would be preferable to handle confounding by stratifying the results be sex, so comparing males in care to males in general population, and females in care to females in the general population.

To clarify, the analysis itself is stratified by sex and sex has also been interacted with care status to allow the four groups (F-gen pop, M-gen pop, F-care, M-care) to have different rate ratios. Therefore, we see this as a question of how to present the findings and not about the actual model itself.

In terms of presentation, yes, there are multiple ways of displaying the results and we have, in previous versions of the paper, used different ways of displaying the effects. What you suggest is

having two reference categories in the model, for example, general population males and females and yes, we would get an easier comparison within sexes. The flipside of that is that we do not get the immediate comparison between sexes. If care experienced males and females are compared to a different baseline, we cannot easily see how different rates ratios for care experienced males are compared to care experienced females (and our results do show a notable difference).

We ended up using the single reference group, general population females, to see how different every other category is from that. The reference group is also one that tends to have the lowest hospitalisations and therefore made logical sense. While you do not get an immediate numeric difference in the tables between general population males and care experienced males, you can see how far apart they are on the figure and that will give you a sense of the difference. However, with two separate baselines, as described above, you could not assess the difference in rate ratios between care experienced males and females from the plot.

Recommendations for analysis and reporting of results:

• I recommend that the authors follow the RECORD guidelines for reporting observational studies, and particularly provide flow diagrams that illustrate which populations are included in the different analyses.

We have used the STROBE checklist as this is the one requested by the journal for observational studies. RECORD is a slight extension of this for linked data studies. Much of this extra information requested in RECORD (such as related to the linkage methods, validation, flow diagram etc.) has been provided in the cohort profile paper referenced (Allik et al 2020 http://dx.doi.org/10.1136/bmjopen-2021-054664).

• I recommend reporting only the results of the current study in your results section and reporting comparisons to other studies in the discussion (e.g., lines 3-7 and 11-14 on page 22).

We have reviewed the manuscript with this in mind.

• When reporting results, I recommend reporting the prevalence of the outcomes in each population group and the risk difference between groups, as well as the relative risk. For example, while the authors report that relative risk of epilepsy among the foster care group compared to the general population group is the highest (see line 59-60 pg5, and line 10 pg8), the risk difference between the two populations is 0.7%. Whilst this equates to a relative risk of 2.4, in absolute terms it means that an additional 7 children out of every 1000 children in the care population have epilepsy compared to every 1000 children of the general population.

For asthma whilst the relative risk is not large, the risk difference between the groups is greater than epilepsy (1.6%) which equates to an additional 16 children out of every 1000 having asthma, compared to every 1000 children in the general population. So, whilst the relative risk makes the difference between groups for epilepsy seem the most substantial, the absolute risk difference and

prevalence estimates show that in terms of actual numbers of children, the increase in asthma in the care group has potentially more impact on service delivery.

Our aim is to compare prevalence and hospitalisation rates between two cohorts and therefore we focus on providing evidence to show these differences. We don't believe presenting the absolute differences in prevalence add much to this paper, especially as these are very easily calculated from the table. The example provided by you highlights that asthma is a much more prevalent condition among children compared to epilepsy or diabetes. The absolute number of children (and absolute differences between population groups) with asthma will be much larger compared to the other conditions.

• I recommend reporting the results of sensitivity analyses in the supplementary material rather than stating "(not shown)" (Line 29 pg 6).

We have added results from the event history models that include sex and age interaction in our supplementary material. However, we have not provided results for other additional analysis as the evidence is not robust. We have kept the references to these analysis/models, as we wish to note to the reader what sensitivity and additional analysis we have undertaken and whether these contributed to our key findings.

• Table 1: I would recommend:

o reporting the descriptive variables unstratified by care group and unstratified by chronic condition. This would provide a reference for the values within these groups, e.g., providing an all-children column, and all children in care and general population columns.

The total population sizes of the cohorts and the descriptive data on these have been previously published in our cohort profile paper. We published the cohort profile paper so that we did not have to repeat all details of the study multiple times. We believe the descriptive data presented here is sufficient for the purpose of this specific research agenda and readers who wish to see more information have open access to the previously published work.

o providing the denominator for the prevalence estimates, for example providing the total number of children in care and general populations.

These have been provided in text in the first paragraph under Data and Methods.

o keeping the decimal places consistent for the hospitalisation means reported (e.g., mean hospitalisations for asthma reported to 1 decimal place).

We have kept the two decimal points to show that there is a small difference in the means between the two populations. If we rounded these to 1 decimal point, this information would be lost.

o For deprivation in the diabetes CEC column, what row do the 17 and 12.0 values correspond to, 1-Low or 2?

Because of statistical disclosure control, we were not able to show the breakdown of children separately for deprivation quintiles 1 and 2 (the cell values were below 10). Therefore, we had to combine these into one group to get the data released from the safe haven. This only applies to data presented in Table 1 and in models we were able to use the exact deprivation quintile. We apologise we forgot to include that note in the table and have added this now.
